# Exploring Disease Management and Control through Pathogen Diagnostics and One Health Initiative: A Concise Review

**DOI:** 10.3390/antibiotics13010017

**Published:** 2023-12-23

**Authors:** Riya Mukherjee, Jasmina Vidic, Sandrine Auger, Hsiao-Chuan Wen, Ramendra Pati Pandey, Chung-Ming Chang

**Affiliations:** 1 Graduate Institute of Biomedical Sciences, Chang Gung University, Taoyuan 33302, Taiwan; riya.mukherjee1896@gmail.com; 2 Master & Ph.D. Program in Biotechnology Industry, Chang Gung University, Taoyuan 33302, Taiwan; 3 Micalis Institute, INRAE, AgroParisTech, Université Paris-Saclay, 78350 Jouy-en-Josas, France; jasmina.vidic@inrae.fr (J.V.); sandrine.auger@inrae.fr (S.A.); 4 Department of Pet Healthcare, Yuanpei University, Hsinchu 300, Taiwan; sjwen@mail.ypu.edu.tw; 5 School of Health Sciences and Technology (SoHST), UPES, Dehradun 248007, Uttarakhand, India; 6 Department of Medical Biotechnology and Laboratory Science, Chang Gung University, Taoyuan 33302, Taiwan; 7 Laboratory Animal Center, Chang Gung University, No. 259, Wenhua 1st Road, Guishan Dist., Taoyuan 33302, Taiwan

**Keywords:** “One Health” approach, pathogen detection strategies, infectious pathogens, zoonotic viral diseases, biosensors

## Abstract

The “One Health” initiative is a critical strategy that recognizes the interconnectedness between human, animal, and environmental health in the spread and containment of infectious pathogens. With the ease of global transportation, transboundary disease outbreaks pose a significant threat to food safety and security, endangering public health and having a negative economic impact. Traditional diagnostic techniques based on genotypic and phenotypic analyses are expensive, time-consuming, and cannot be translated into point-of-care tools, hindering effective disease management and control. However, with advancements in molecular methods, biosensors, and new generation sequencing, rapid and reliable diagnostics are now available. This review provides a comprehensive insight into emergent viral and bacterial pathogens and antimicrobial resistance, highlighting the importance of “One Health” in connecting detection and effective treatment. By emphasizing the symbiotic relationship between human and animal health, this paper underscores the critical role of “One Health” initiatives in preventing and controlling infectious diseases.

## 1. Introduction

The “One Health” approach is a concept that aims to achieve the best possible health outcomes by recognizing the interconnectedness among people, animals, plants, and their shared environment at the local, national, and global levels. This concept, initiated in the early 2000s, follows the resurgence and emergence of infectious diseases. Drivers such as agricultural practices, globalization, and the wildlife trade provide multiple opportunities for pathogens to evolve into new forms, making spillover events from animals to humans more frequent and intense. Numerous contemporary health issues, including the proliferation of zoonotic infectious diseases, environmental pollutants, antimicrobial resistance, shifts in food systems driven by climate and market forces that affect food and feed supplies, and challenges related to malnutrition, including obesity, are interconnected within the realms of humans, animals, and the ecosystems that form their environment. The extraordinary One Health approach can inspire scientists to develop new areas of research to generate innovative ideas by carrying out interdisciplinary work that combines biology, ecology, mathematics, economics, and social sciences and experimenting with new systems that respect all dimensions of health. Zoonotic diseases represent a major public health worry, as more than 70% of newly emerging diseases are transferred from animals to humans, and 60% of human infectious diseases are shared with animals [1]. This implies that zoonotic diseases have been implicated in recent outbreaks such as the Ebola and coronavirus pandemics, as well as notable cases of foodborne illnesses. The transmission of these diseases can occur not only through direct contact with animals or vectors or the consumption of animal products but also through the intake of contaminated vegetables cultivated in regions where domestic or wild animal manure or irrigation water is utilized [2,3]. The World Health Organization (WHO) states that most foodborne diseases result from infections, wherein various bacteria, viruses, or parasites enter the body through the consumption of contaminated food [4]. Moreover, antimicrobial resistance stands as a pressing and intricate global health challenge with multifaceted dimensions. The growing apprehension centers around the rise of multidrug-resistant superbugs, causing infections that are challenging to treat with existing antimicrobial agents. This resurgence harkens back to the pre-antibiotic era, sparking worries about the potential onset of a post-antibiotic era. Tackling this pressing threat demands the execution of a comprehensive strategy outlined in recent years. Successful implementation will necessitate unwavering political commitment, investment in systems and research, and a One Health approach that fosters enhanced communication, cooperation, and collaboration among diverse professional disciplines and organizations crucial at the nexus of human, animal, and environmental health [5]. With the rise of global health threats and increased international transportation, the risk of transboundary disease outbreaks has grown. The current regulatory framework recognizes the benefits of employing a One Health strategy to control and eliminate infectious zoonotic diseases or outbreaks. The demand for swift and dependable diagnostics is urgent, especially in the face of emerging and recurring infectious challenges. Recent progress in molecular techniques, biosensors, and next-generation sequencing presents unparalleled opportunities for more efficient and accessible disease detection. Grasping and utilizing these technologies is essential for effective disease management [6,7]. The One Health Initiative underscores the interconnectedness of human, animal, and environmental health, yet the existing literature may lack a thorough examination of the environmental aspect. It is crucial to delve into the role of environmental factors in disease transmission and their implications for diagnostics and management, a fact that demands more attention. The current knowledge base falls short in adequately addressing the nuanced socioeconomic impacts of infectious diseases and how One Health strategies contribute to resilience. There is a notable gap in exploring the broader societal implications and the effectiveness of One Health interventions in mitigating economic consequences. While molecular methods, biosensors, and sequencing technologies are mentioned, there appears to be a shortfall in critically evaluating these diagnostic tools. A thorough assessment of their strengths, limitations, and practical applicability is vital for guiding future research and implementation. A comprehensive review can illuminate how One Health approaches integrate diagnostics, addressing not only the detection of pathogens but also the escalating challenge of antimicrobial resistance.

## 2. Methodology

We extensively reviewed the literature, concentrating on disease management, pathogen diagnostics, and the One Health Initiative. We identified crucial studies and articles from reputable sources at the crossroads of these subjects. To amass pertinent peer-reviewed articles, we utilized esteemed academic databases like PubMed, Scopus, and Web of Science. Employing search terms such as “disease management,” “pathogen diagnostics,” and “One Health Initiative” ensured a thorough exploration. We amalgamated information gathered from the literature to discern recurring themes, hurdles, and advancements in managing and controlling diseases through pathogen diagnostics within the One Health Initiative framework. By integrating these findings, we forged a comprehensive understanding of how pathogen diagnostics and the One Health Initiative synergistically contribute to effective disease management and control strategies.

## 3. Current Landscape of Disease

**Figure 1:**
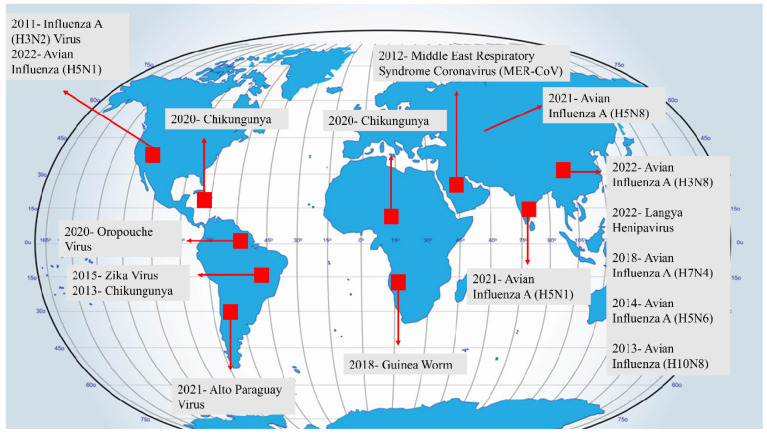
Emerging diseases across the globe.

Infectious diseases limit productivity and result in significant economic losses in each sector. Transboundary animal diseases (TADs) are economically important, have global reach, and require management. TADs can have significant implications for food security. Food-borne pathogens comprise microorganisms such as bacteria, viruses, and fungi, as well as parasites that cause food spoilage and infection [8]. Food-borne pathogens are a major threat to food safety, as they can cause human diseases if animal products infected with toxins are consumed [9]. Previous studies have concluded that 66%, 4%, and 26% of food-borne diseases have developed from bacteria, viruses, and chemicals, respectively [10]. In the past, diseases like tuberculosis, polio, smallpox, and diphtheria were widespread, causing significant illness and death before the introduction of vaccines [11]. Simultaneously, animal diseases like rinderpest spread through trade routes and military movements, leading to devastating consequences for livestock and the communities relying on them. In the 21st century, we have experienced a series of significant infectious disease outbreaks, with the COVID-19 pandemic being particularly devastating, impacting lives and livelihoods worldwide. Other notable instances include the 2003 SARS outbreak, the 2009 swine flu pandemic, the 2012 MERS outbreak, the 2013–2016 Ebola epidemic in West Africa, and the 2015 Zika virus epidemic. These events have caused considerable illness and death, crossing borders to affect populations in numerous countries [11]. Over the last two decades, advancements in medicine, improved access to healthcare, and better sanitation have reduced the overall impact of infectious diseases, particularly in terms of lower respiratory tract infections and diarrheal diseases. The rapid development of the SARS-CoV-2 vaccine highlights the effectiveness of modern science in swiftly addressing emerging pathogen threats. However, infectious disease challenges persist in countries with lower incomes, and neglected tropical diseases, HIV infection, tuberculosis, and malaria continue to cause significant mortality and morbidity. Additionally, deaths from emerging and re-emerging infections, distinct from seasonal and endemic infections, have persisted throughout the twenty-first century. This suggests a potential new era of infectious diseases characterized by outbreaks involving emerging, re-emerging, and endemic pathogens that spread rapidly, facilitated by global connectivity and shifting ranges due to climate change [12,13]. The emergence of diseases stems from intricate interactions between microbes and humans, often influenced by a variety of complex factors. For instance, the movement of populations can lead to the expansion of a once localized infection into a city with inadequate public health infrastructure, facilitating its establishment in the broader population. Subsequently, the city may serve as a point of origin for further transmission. Key contributors to disease emergence include microbial adaptation and change, ecological shifts, human demographics and behavior, advancements in technology and healthcare, travel, trade, and industrial activities, breakdowns in public health measures, and varying levels of susceptibility to infection (Figure 1) [14,15].

### 3.1. Zoonotic Viral Pathogens

Exposure to meat from an infected animal can lead to zoonotic food-borne infections. Although this type of transmission is a valid concern, it is the least common method of virus transmission [16]. Most zoonotic infections are primarily acquired through direct contact with an infected animal, which can result in cutaneous lesions at the point of contact. However, there is also evidence to suggest that certain cases have occurred through mucosal pathways or after contact with infected surfaces (fomites). Animals, particularly wild animals, are thought to be the source of more than 70% of all new illnesses in humans [17]. In recent decades, the chikungunya virus, human immunodeficiency virus type 1, Ebola virus, hantavirus pulmonary syndrome virus, Hendra virus, Nipah virus, severe acute respiratory syndrome (SARS), and coronavirus (COVID-19) are examples of viruses that have caused emergent diseases in humans [18,19,20]. The transportation of companion animals that are afflicted could cause the poxvirus to be released into a new habitat, which is a probable scenario for a future outbreak. The current understanding of the epidemiology of poxvirus points to the need for more effective detection and management of these infections. At least three genera of poxviruses, including orthopoxvirus and parapoxvirus, contain zoonotic poxviruses. Food-related incidents have been linked to the transmission of (SARS), monkeypox, norovirus and the Ebola virus [21]. Foodborne viruses enter the host organism through the gastrointestinal tract and reproduce in the intestinal tract before spreading throughout the body via the lymph nodes. Thus, the pathogenicity of the entering virus is influenced by its survival in the harsh, acidic environment of the stomach and proteolytic enzymes in the intestinal system.

The ‘gold standard’ for the detection of the majority of viruses is the polymerase chain reaction (PCR) method, which is rapid (a few hours to provide results) and highly specific. Real-time reverse transcriptase-PCR (RT-PCR) and qRT-PCR, which enable viral RNA detection, are of great interest because of their effectiveness needed for the efficient prevention of infection spreading. Both methods are equally effective in the detection of bacterial pathogens [22].

Hepatitis E virus (HEV) is prevalent on domestic swine farms around the world, and it can infect pigs of all ages. As a result, the majority of foodborne HEV outbreaks have been linked to pork liver and pork liver-containing products [23,24]. HEV outbreaks have also been linked to other foods, including cow milk and the meat of wild animals. HEV typically manifests as delimiting acute hepatitis in high-risk populations. Moreover, persistent hepatitis and extrahepatic symptoms may occur [23]. The diagnosis of HEV infection can be performed through direct detection of the viral biomarker or indirect detection based on the host’s immune response to HEV. The direct tests detect parts of the viral particles, such as HEV ribonucleic acid (RNA) or viral capsid antigens. They usually have high specificity and low sensitivity [25]. In contrast, the indirect tests targeting anti-HEV antibodies in blood have high sensitivity but low specificity. Consequently, diagnostic testing for suspected patients is usually performed by combining serology and nucleic acid amplification testing. The new generation of available HEV diagnostic tests is of advanced performance, but tests are still not standardized, and in many parts of the world, no diagnostic kit for commercial usage is available. This prevents the efficient control of the spread of the disease. Aquatic wild birds constitute the main reservoir for avian influenza virus (AIV). These viruses represent a global threat to animal health and the poultry industry and may cause a zoonotic infection that has effectively transcended the host organism threshold to infect humans [26]. There is particularly high concern for pandemic emergences, which may have serious consequences on human health and cause enormous economic losses. AIVs are divided into low and highly pathogenic strains regarding their pathogenicity in chicken. The highly pathogenic AIV (HPAIV) causes systemic infections and results in a high mortality rate. The infection of poultry with low pathogenic avian viruses (LPAIV) generally leads to mild clinical signs, while in waterfowl species, often no clinical signs are invoked. Certain subtypes can change from low to high pathogenicity [8], as in the case for LPAIV strains of the H5 and H7 subtypes, which acquired a highly pathogenic phenotype during infections in avian species. HPAIVs have killed both domestic and wild birds and have led to the destruction of hundreds of millions of domestic birds (e.g., around 30 million chickens were killed in The Netherlands, while 0.8 million were killed in France during the AIV outbreaks in 2003 and 2017, respectively) [27]. South Korea faced an H5N6/H5N8 outbreak, which paralyzed its poultry industry; 20 million birds have been killed in January 2017. Such pandemic HPAIVs are difficult to control because no tool for in-field diagnostics is available. Samples collected by veterinarians at farms are first transported to authorized laboratories where the diagnosis of an HPAIV strain takes at least a whole day. To contain and eradicate zoonotic influenza viruses, researchers must not only conduct strategic virus surveillance in both animal and human populations but also gain a better understanding of the obstacles that a virus must overcome to cross the species barrier and infect humans. The influenza pandemic of 1918-1919, also known as the Spanish flu, caused widespread sickness and resulted in an estimated 40 million deaths worldwide. Existing studies have revealed that the pandemic virus contained genes that were derived from avian-like influenza virus strains. This virus is the common ancestor of both human and classical swine H1N1 influenza viruses. Pigs are believed to have played a role in this process, as they can be infected with both avian and human virus strains, which has resulted in various reassortants being isolated from them [28].

**Figure 2:**
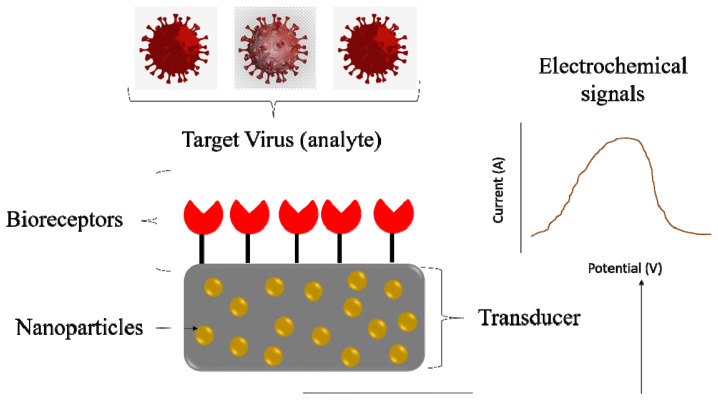
Biosensors based electrochemical signal for virus detection.

Standard diagnostic methods based on virus propagation and isolation from embryonated chicken eggs are effective and sensitive, but they are time consuming and require complex procedures for sample collection and handling. Molecular methods based on RT-PCR need extracted genetic material. Portable later-flow devices proposed for some viral disease diagnostics are not multiplex and lack sensitivity [5]. There is strong interest in developing new point-of-care biosensing systems for the early detection of viral diseases with high sensitivity and specificity. Moreover, a new version of the RT-PCR assay has been developed in accordance with the One Health program to meet the criteria of multi-species origin IAV detection. The matrix protein area is thought to be the most important aspect for detecting all AIV subtypes; however, given the amount of genetic drift that occurs over time, changes to this matrix protein region have added novelty to this assay [29]. Rapid and robust virus detection methods could significantly help in future pandemics. Biosensors for virus detection based on electrochemical, plasmonic, and optical signals make them ideal platforms for virus detection (Figure 2). Combining such portable devices with suitable nanomaterials can enhance the portfolio of available diagnostic kits. Biosensors’ sensitivity and selectivity are usually connected with the recognition element (antibody, DNA probe, aptamer) changing electronic or optical properties in the presence of the targeted virus. Biosensor strategies can be readily adopted for the detection of new emerging zoonotic viruses.

### 3.2. Antimicrobial Resistant Pathogens

The rise of antimicrobial resistance (AMR) is due to the misuse and overuse of antibiotics in both humans and veterinary and agricultural practices [30]. The World Organisation for Animal Health (OIE), along with the Food and Agriculture Organization of the United Nations (FAO) and the World Health Organization (WHO), consider antibiotic resistance to be a major priority. Managing this issue requires coordinated and concerted efforts across multiple sectors, including animal and agricultural production, food processing, human health, and the environment. The non-rational use of veterinary antibiotics may result in the pressure selection of resistant pathogens. While increasing AMR awareness is critical, new antibiotics and therapy techniques must also be developed. The One Health platform raises public awareness about AMR by implementing a dual AMR track. To gain a new perspective, an algorithm was developed as a tool to quickly assess the potential for a new or emerging livestock disease to harm humans through the consumption or handling of meat products, so that the risks and uncertainties can be understood, and appropriate precautions and policies can be enacted. The One Health systematic method of assessing AMR will aid in a better understanding of antibiotic resistance [31]. The surveillance of AMR, legislative reforms, new economic models, diagnostics and detections, and alternative techniques to combat resistant infections should all be considered for a rapid response to this issue. Antibiotic stewardship refers to efforts to enhance the use of various antibiotics to prevent needless antibiotic use [31]. There is a variety of options for the use of antibiotics, including the use of bacteriophages and immune modulators [32]. The sequence of events from the onset of the disease in cattle to the discharge of the causative agent from an infected animal, the contamination of fresh meat, and possible harmful consequences in humans following contact with meat was developed using an algorithm. The concept of One Health has long been a component of human civilization. The One Health approach can grasp the interconnectedness and inherent complexities of human and animal health and the environment by addressing their relationship. This extraordinary method may inspire scientists to develop new fields of research to generate innovative ideas. Science has demonstrated its ability to successfully integrate all sectors to identify a path for detecting emerging diseases and pathogens, as well as developing novel therapeutic procedures [1,2].

The prevalence of organisms exhibiting AMR, especially resistance to multiple antibiotics, shows large variations in the percentages of AMR depending on the microorganism, antimicrobial agent, and geographical region [33]. Most of the ESKAPE pathogens (Enterococcus faecium, S. aureus, Klebsiella pneumoniae, Acinetobacter baumannii, Pseudomonas aeruginosa, and Enterobacter species) are multidrug resistant isolates, and one of the greatest challenges in clinical practice. Initially, the ESKAPE bacteria surveillance was focused on healthcare-associated infections. Today, the increasing AMR awareness of ESKAPE strains has led to extensive investigations in various ecosystems. The presence of multi-resistant ESKAPE strains carrying mobile genetic elements and gene cassettes encoding resistance to antibiotics or biocides in the water cycle is not demonstrated. Both freshwater and marine systems act as a sink for ESKAPE bacteria that enter aquatic systems through treated and untreated sewage, hospital waste, and agricultural run-off [34]. Water contamination with AMR bacteria, especially water co-contaminated with antibiotic residues, may induce resistance in autochthonous bacteria through horizontal gene transfer as well as through spontaneous de novo point mutations, which can change the cellular targets of antibiotics or the expression of resistant genes, leading to increased antibiotic resistant of the bacteria [35]. In horizontal gene transfer, genetic material can transfer between related or unrelated species via mobile elements. Given the potential for transmission and environmental dissemination, ESKAPE infections, which are well known for their antibiotic resistance in human healthcare, indirectly connect with the One Health paradigm. Antibiotic-resistant bacteria can be identified in animals and the environment, while being largely linked with humans, demonstrating how interrelated humans, animals, and environmental health are [36,37,38].

## 4. Advancements in Diagnostics for Disease Management

Infectious diseases caused by various pathogens can have devastating effects on public health. Although vaccines, antimicrobial drugs, and antiviral drugs are available, their development requires lengthy clinical trials, which can delay the effective control of infectious diseases. In the absence of specific drugs, selecting appropriate detection techniques for identifying specific organisms can be a more efficient way to deal with infectious diseases. This can help improve the effectiveness of treatment and reduce the spread of infections, leading to a prompt response to serious public health events. The identification of sources and pathways of aquatic ecosystem contamination and the accessibility of a fast, accurate method to warn about the critical level and spread of AMR bacteria in the water cycle will enable the reduction of AMR bacterial pathogens at the source. Conventional methods for the detection of AMR bacteria require long protocols and highly trained personnel [34]. Currently, the methods of plating, culturing, and gene sequencing are prevalent in practices for determining the drug-resistance status of infectious agents, but some biosensors have started to be proposed [35]. Most countries have developed AMR national action plans. However, the effective implementation of action plans and multisectoral collaboration for the containment of AMR still must be improved. Moreover, the global action plan for the containment of AMR can be realized only through a multidisciplinary and multidimensional approach at the human–animal–environment interface. Some conventional detection techniques include microbial culture, hemagglutination inhibition tests, and enzyme-linked immunosorbent assays (ELISAs) [39]. Of these, pathogenic microorganisms can be challenging to identify based on the morphological characteristics alone, which can lead to low specificity and sensitivity (Table 1). Immunological methods such as hemagglutination inhibition assays and ELISAs are simple to perform, but they have drawbacks such as high false positives, cost, and poor thermal stability. However, molecular diagnostic techniques focused on nucleic acid detection have revolutionized the diagnosis of infectious diseases, with a short turnaround time and high sensitivity [40]. As a result, the diagnosis and treatment of parasitic diseases have undergone significant changes due to increased awareness of their different clinical manifestations. Recent advancements in diagnostic techniques have revolutionized the way we detect and treat parasitosis caused by metazoans and protozoa. With the help of these techniques, we can now diagnose the infection more quickly and accurately, leading to a better chance of successful treatment (Table 2). Moreover, the understanding of parasite biology and the development of drug screening tools have opened new doors for identifying potential drug targets and antiparasitic molecules. It is crucial to note that diagnostic and treatment measures should not only be aimed at infected humans but also at other vertebrate hosts (reservoirs) involved in the transmission of zoonotic diseases. This approach aligns with the One Health concept, which emphasizes the importance of the collaborative effort of multiple disciplines to achieve optimal health for people, animals, and the environment [41,42]. Traditional diagnostic methods, relying on genotypic and phenotypic analyses, have proven costly, time-intensive, and impractical for point-of-care applications (Table 1).

### Significance of Novel Detection Strategies for Infectious Pathogens

There are various novel detection strategies for pathogens which are of paramount importance in addressing emerging infectious diseases (Table 3). The methods often offer faster and accurate identification of pathogens, allowing for early diagnosis and the prompt initiation of treatment. Many of the strategies are highly specific and sensitive, enabling the differentiation of closely related pathogens and the detection of low pathogen concentrations [39]. Various methods enable the healthcare systems and public health agencies to monitor the prevalence and distribution of pathogens, helping to identify outbreaks and implement timely control measures. The accurate identification of the causative agent of an infectious disease helps healthcare providers to prescribe accurate treatments, reducing the unnecessary use of antibiotics and other medications. The RT-PCR assay is a universal technique that can be employed for detecting a range of infectious pathogens and can also be used to detect antimicrobial resistance. ELISA, a plate-based approach, and traditional pathogen detection methods based on culturing are time-consuming and usually fail to distinguish between pathogenic strains. As a result, molecular techniques like PCR and RT-PCR are mainly employed for diagnosing and identifying infectious diseases in animals. On the other hand, next-generation sequencing (NGS) is applied in the genetic studies of pathogenic agents. The new generation of high-throughput sequencing allows for the parallel analysis of billions of nucleotides in one short and affordable way. NGS is more and more accessible to many laboratories, enabling the extensive monitoring of epidemic pathogen spreading and AMR analyses [39,40]. Whole-genome sequencing (WGS) is especially recommended for pathogen diagnostic and surveillance by food safety regulatory agencies. NGS can also detect a huge range of pathogens and antimicrobial resistance. The most interesting aspect about NGS is that it can detect the specific genes responsible for showing resistance as well.

Aside from these tactics, biosensing technologies for identifying animal diseases with high sensitivity and specificity are increasingly under development [41,42]. Recent advances in point-of-care diagnostic kits that involve novel nanotechnologies have been extensively investigated in the fields of pathogen sensing and food safety. For instance, employing graphene, graphene oxide, and other carbon-based nanomaterials, gold nanoparticles, and molecularly imprinted biosensors have been well-publicized, particularly for their ability to produce massive signal augmentation and amplification with precise selectivity [42]. Exploring novel nanomaterials like black phosphorus would be advantageous because of their fascinating qualities, such as direct bandgap, strong structural and functional anisotropy, high conductivity, and electron transfer capacity, which could boost detection sensitivity greatly [43,44].

## 5. One Health Initiatives for Infectious Disease Management and Control

A One Health approach is crucial for the effective prevention and control of zoonotic diseases. This framework can be implemented at various levels, including local, sub-national, national, regional, or international. By applying a One Health approach, zoonotic disease prevention and control programs can be optimized, leading to more efficient use of resources, such as finances, infrastructure, and personnel. This can ultimately improve the quality and timeliness of healthcare delivery, potentially saving lives. Despite increasing awareness of the One Health approach, lack of communication and coordination between human health, animal health, and environment sectors can still hinder successful implementation. The organizations, which include the Food and Agriculture Organization of the United Nations (FAO), the World Organisation for Animal Health (OIE), and the World Health Organization (WHO), have effectively utilized a multisectoral One Health approach [45,46]. This approach involves mandated inter-agency collaboration and endorsement of One Health to promote sustained collaboration for zoonotic disease control at all levels, including local, subnational, national, regional, and international. Table 4 provides a summary of One Health policy interventions addressing various pathogens. It includes information about the targeted pathogens, key policy components, and approaches employed. It is crucial to emphasize the importance of vaccines since many infectious diseases can spread from animals to humans, and those that affect livestock and wildlife can also impact human health, food production, and social stability. Given their ability to prevent the spread of disease among humans and animals and their shared habitats, vaccines are essential components of the One Health agenda. They can limit the spread of disease as an intervention as well as prevent the onset of disease as a prophylactic approach. In fact, several vaccines are made specifically for domesticated animals and cattle to prevent diseases that affect both humans and animals, such as leptospirosis, rabies, and Rift Valley Fever [47].

In 2006, Egypt faced a serious threat to public health and the poultry industry in the form of an outbreak of avian influenza H5N1. The government’s swift action and collaboration with international organizations through the implementation of a One Health initiative proved to be highly successful in controlling the spread of the disease. The use of real-time PCR for pathogen diagnostics played a critical role in the early detection and diagnosis of the disease. This technology was utilized to detect the virus in both human and animal samples, enabling the rapid identification and isolation of infected individuals and flocks. Furthermore, it was instrumental in monitoring the genetic evolution of the virus, providing valuable information for vaccine development [48,49]. The success of this initiative serves as a model for future disease outbreaks and highlights the importance of international collaboration in addressing global health challenges. The One Health initiative exemplified an impressive level of collaboration among multiple ministries and international organizations, including the Ministry of Health, Ministry of Agriculture, and Ministry of Environment, as well as the World Health Organization and the Food and Agriculture Organization of the United Nations. The initiative’s swift implementation of measures, such as culling infected birds, vaccinating poultry, and increasing public awareness through education campaigns, led to a successful containment of the outbreak within a few months, resulting in a significant decrease in human cases [48]. Since 2009, no new cases have been reported, and the poultry industry has been able to make a full recovery due to the implementation of increased biosecurity measures and vaccination programs.

Effective management of infectious disease outbreaks is crucial, and this case study showcases the importance of pathogen diagnostics and One Health initiatives in achieving this. By bringing together the human and animal health sectors and utilizing advanced diagnostic technologies for early detection and diagnosis, we can successfully manage and control disease outbreaks. A prime example of this is the successful handling of a H5N1 outbreak in Egypt [50].

**Table 4:** Overview of One Health policy interventions.

Intervention	Pathogens Addressed	One Health Policy Approaches	Ref.
Integrated surveillance systems	Bacteria, viruses, parasites	Establishing coordinated surveillance systems that capture human, animal, and environmental data. Integration of data across sectors for a comprehensive view.	[51]
Zoonotic disease control Policies	Zoonotic pathogens	Development and implementation of policies focused on controlling and preventing the spread of zoonotic diseases. Includes vaccination programs, biosecurity measures, and regulations on animal trade.	[51,52]
Antimicrobial resistance (AMR) policies	Bacteria, fungi	Policies aimed at regulating and promoting responsible use of antimicrobials in human and veterinary medicine. Also addressing the environmental aspects of antimicrobial resistance.	[53]
One Health research funding	Various pathogens	Allocating research funds to interdisciplinary studies that investigate the interconnectedness of human, animal, and environmental health. Encouraging collaborative research initiatives.	[53]
Education and capacity building	Various pathogens	Implementing educational programs to raise awareness about One Health principles. Building capacity among professionals in human and veterinary medicine, as well as environmental sciences.	[54]
Environmental conservation policies	Pathogens in the environment	Policies focusing on habitat conservation, sustainable land use, and water management to reduce the risk of disease transmission from wildlife to humans and domestic animals.	[55]

## 6. Discussion

A paradigm shift in how we approach illness management and control is represented by the One Health Initiative. The One Health concept offers a comprehensive framework for solving complicated health concerns by recognizing that illnesses can cross species borders and that human activities have an impact on ecosystem health. The One Health Initiative’s capacity to create collaboration is one of its main advantages. The knowledge, information, and resource sharing that is encouraged by this multidisciplinary approach is crucial for efficient disease surveillance, prevention, and control [51]. The findings we reached about the viability of cross-disciplinary collaboration initiatives among experts in human, animal, and environmental health are in line with current worldwide policy recommendations for the improved horizontal integration of such initiatives. Therefore, identifying infectious disease risk to human and animal populations could be aided by integrated disease surveillance. But when viewed as a whole, our analysis emphasizes the necessity of additional extensive, tightly monitored comparative trials of One Health illness prediction and control methods. Such studies would offer more convincing proof of the advantages of broad methods. Larger implementation studies of surveillance systems incorporating data from people, animals, and the environment should be conducted specifically. As these systems are put into place, their efficacy should be compared to that of more segregated systems. The comparative effectiveness of the One Health approach over single-sector efforts or projects that focus only on human and animal health and ignore the environmental and ecosystem factors underlying the problem could also be more clearly established in larger controlled intervention trials of One Health approaches for the control of several infectious and chronic diseases [56].

The One Health Initiative relies heavily on pathogen diagnostics. They operate as the starting point for the early identification, surveillance, and early detection of infections that may affect both human and animal populations. The early identification of diverse zoonotic pathogens is crucial for prompting effective responses. Traditional diagnostic approaches provide a fundamental framework for disease management within the One Health paradigm and its well-established procedures, such as culture-based microbiology and serological tests. They offer historical information on the prevalence and spread of pathogens, making it easier to track changes in disease patterns over time. Traditional approaches can act as validation and reference standards, guaranteeing the precision and dependability of more recent diagnostic techniques. Furthermore, they are essential for disease surveillance and control in vulnerable populations in resource-constrained contexts where modern technology may not be easily available. By teaching local practitioners’ fundamental diagnostic abilities, these traditional tools also aid in capacity building. On the other hand, novel diagnostic approaches offer amazing capabilities that are essential in the One Health environment and are fueled by technology advancements in molecular biology, genomics, proteomics, and bioinformatics. They are exceptional at early disease detection and identification, allowing for quick reactions to impending epidemics. When dealing with the complicated dynamics of disease transmission in complex ecosystems, these technologies frequently enable high-throughput testing and the simultaneous identification of many pathogens. In addition, modern diagnostics, in particular genomics, enable thorough genetic epidemiology studies that aid in identifying disease evolution, unraveling disease transmission networks, and informing focused intervention methods. Fundamentally, the integration of conventional and cutting-edge diagnostic techniques ensures a comprehensive and successful approach to disease management and control within the One Health framework, eventually protecting the wellbeing of people, animals, and the environment.

## 7. Conclusions and Perspectives

Emerging viral or bacterial pathogens have the potential to impose substantial mortality, morbidity and economic burdens on human populations. The mutual dependence of human and animal health is central to the One Health initiative as an integrated strategy for infectious disease control and management. The historical record of IAV outbreaks, as well as many other viral outbreaks and constant bacterial infections from food ingestion, have indicated that novel epidemics or pandemic strains can emerge unexpectedly from a previously unknown pathogenic population. The bioinformatics monitoring of any such epidemiologically relevant strains remains critical in terms of potential qRT-PCR sensitivity repercussions. Therefore, sensitive and specific detection of the widest possible spectrum of strains and subtypes is crucial. In conclusion, pathogen diagnostics are vital weapons in the armory of the One Health Initiative. They enable us to adopt a proactive approach to emerging infectious illnesses, zoonotic risks, and antibiotic resistance by facilitating early identification, surveillance, and data-driven decision-making. Adopting a One Health perspective and utilizing the potential of pathogen diagnostics can improve disease management and control, eventually protecting both human and environmental health. Governments, organizations, and stakeholders from all over the world must prioritize and invest in these important aspects of contemporary disease control programs.

## Figures and Tables

**Table 1:** Conventional diagnostic techniques and their limitations.

Pathogen Type	Diagnostic Technique	Limitations	Ref.
Viruses	Serological assays	Limited to past infection/exposure	
	Viral culture	Slow and requires specific growth conditions	[40]
	Antigen-based assays	Sensitivity may vary with the test	
Bacteria	Culture-based techniques	Slow results	
	Gram-staining	Limited to bacterial cell structure	[40]
	Biochemical tests	Species-level identification may be lacking	
Fungi	Culture-based techniques	Slow growth and identification	
	Microscopic examination	Limited to visual characteristics	[40]
	Serological tests	Limited sensitivity and specificity	
Parasites	Microscopic examination	Limited to detecting visible stages	
	Serological tests	May not detect early infections	[40]
	Stool examination	May require multiple samples	

**Table 2:** Novel detection strategies and One Health interventions.

Pathogen Type	Novel Detection Strategy	One Health Intervention	Importance/Significance	Ref.
Bacteria	Molecular diagnostics (PCR)	Early disease surveillance in animals and humans	Rapid and specific detection for timely intervention and understanding genetic factors in disease transmission.	[40,41]
	Next-generation sequencing	Cross-sector data sharing and collaboration		
Viruses	Metagenomic sequencing	Integrating environmental data	Detecting emerging viruses and understanding reservoirs and potential for point-of care testing and rapid response.	[40,41]
	CRISPR-based diagnostics	Educating healthcare professionals		
Fungi	DNA barcoding	Monitoring wildlife populations	Identifying fungal pathogens in zoonotic diseases and rapid identification of fungal species.	[40,41]
	MALDI-TOF mass spectrometry	Promoting hygiene and sanitation in food production		
Parasites	Nucleic acid amplification	Establishing One Health policies	Improved diagnosis of parasitic infections and identifying and tracking zoonotic parasites.	[40,41]
	Serological tests with antigens	Cross-species surveillance		

**Table 3:** Novel detection strategies for detecting pathogens and antimicrobial resistance.

Pathogen Detection Strategy	Subtypes	Advantages	Disadvantages	Pathogens Detected	Antimicrobial Resistance	Ref.
Molecular diagnostics	PCR	Rapid, sensitive, specific, high-throughput, can detect low levels of pathogens.	Expensive, requires trained personnel and specialized equipment.	Bacteria, virus, fungi, parasites	Yes	[41,42]
	Loop-mediated isothermal amplification	Rapid, sensitive, specific, low-cost.	Limited multiplexing capability, susceptibility to non-specific amplification.			
	Nucleic acid sequence-based amplification	Rapid, sensitive, specific.	Limited multiplexing capability.			
Biosensors	Optical biosensors	Rapid, portable, real-time detection, high sensitivity, low sample volume required.	Limited multiplexing capability, may require specialized equipment.	Bacteria, virus, fungi, parasites	Yes	[43]
	Electrochemical sensors	Rapid, portable, real-time detection, high sensitivity, low sample volume.	Limited multiplexing capability.			
	Piezoelectric biosensors	Rapid, sensitive, specific, real-time detection, label-free detection.	Limited multiplexing capability.			
Next-generation sequencing	Whole-genome sequencing	High-throughput, comprehensive pathogen detection and characterization, can identify new and emerging pathogens.	Expensive, requires specialized equipment and trained personnel.	Bacteria, virus, fungi, parasites	Yes	[44]
	Metagenomic sequencing (MGS)	High-throughput, comprehensive pathogen detection and characterization, can identify new and emerging pathogens, can detect co-infections and mixed infections.	Expensive, requires specialized equipment and trained personnel.			
	Targeted amplicon sequencing (TAS)	Rapid and sensitive detection of specific pathogens or gene targets, can detect low levels of pathogen, high throughput with multiplexing capability.	Expensive, requires specialized equipment and trained personnel.			

## Data Availability

Not applicable.
